# The role of autophagy in colorectal cancer: Impact on pathogenesis and implications in therapy

**DOI:** 10.3389/fmed.2022.959348

**Published:** 2022-09-07

**Authors:** Eglal Mahgoub, Jalal Taneera, Nabil Sulaiman, Maha Saber-Ayad

**Affiliations:** ^1^College of Medicine, University of Sharjah, Sharjah, United Arab Emirates; ^2^Sharjah Institute for Medical Research, University of Sharjah, Sharjah, United Arab Emirates; ^3^Baker Heart and Diabetes Institute, Melbourne, VIC, Australia; ^4^Faculty of Medicine, Cairo University, Giza, Egypt

**Keywords:** colorectal cancer, autophagy, tumor microenvironment, endothelial cells, hypoxia, oxidative stress, targeted therapy, MSI-H

## Abstract

Colorectal cancer (CRC) is considered as a global major cause of cancer death. Surgical resection is the main line of treatment; however, chemo-, radiotherapy and other adjuvant agents are crucial to achieve good outcomes. The tumor microenvironment (TME) is a well-recognized key player in CRC progression, yet the processes linking the cancer cells to its TME are not fully delineated. Autophagy is one of such processes, with a controversial role in the pathogenesis of CRC, with its intricate links to many pathological factors and processes. Autophagy may apparently play conflicting roles in carcinogenesis, but the precise mechanisms determining the overall direction of the process seem to depend on the context. Additionally, it has been established that autophagy has a remarkable effect on the endothelial cells in the TME, the key substrate for angiogenesis that supports tumor metastasis. Favorable response to immunotherapy occurs only in a specific subpopulation of CRC patients, namely the microsatellite instability-high (MSI-H). In view of such limitations of immunotherapy in CRC, modulation of autophagy represents a potential adjuvant strategy to enhance the effect of those relatively safe agents on wider CRC molecular subtypes. In this review, we discussed the molecular control of autophagy in CRC and how autophagy affects different processes and mechanisms that shape the TME. We explored how autophagy contributes to CRC initiation and progression, and how it interacts with tumor immunity, hypoxia, and oxidative stress. The crosstalk between autophagy and the TME in CRC was extensively dissected. Finally, we reported the clinical efforts and challenges in combining autophagy modulators with various cancer-targeted agents to improve CRC patients’ survival and restrain cancer growth.

## Introduction

Colorectal Cancer (CRC) is counted as one of the most predominant cancers in both genders with high death rates. CRC is third in terms of prevalence which accounted for 6.1% of new cases and second in terms of the cause of death which accounted for 9.2% of deaths by cancer worldwide (1). There is a high incidence of colorectal cancer at young age (15–39 years) which was estimated by 70.2–82.9 thousand cases in 2019 with a mortality rate of 26.2–30.5 thousand in the same year (2). By the year 2035, it is estimated that the total number of deaths will increase by 71.5 and 60% from colon and rectal cancers, respectively (3). CRC is a heterogeneous disease with numerous variations in its molecular profiles, clinical manifestations and prognosis. CRC prognosis depends on the tumor staging at the time of diagnosis. Currently, the best therapeutic option for stage I and most of the stage II CRC patients is the aggressive surgical resection of the primary tumors which showed high success rates, with/without adjuvant radio-chemotherapy for high risks patients in stage II and stage III of CRC. Notably, stage III CRC patients usually suffer from recurrent disease, which may be associated with micro-metastasis. Stage IV CRC represents a metastatic state with a high risk of relapse and with less/no benefit from surgery. Instead, chemotherapy combinations are usually used at this stage, such as oxaliplatin/irinotecan and folinic acid, 5-fluorouracil (5-FU)-based regimens (4, 5). However, adjuvant treatment is highly accompanied by drug resistance, and ultimately disease progression in metastatic CRC. Recent advances in cancer-targeted therapy as second-line treatment of CRC in combination with chemotherapy, to disrupt signaling pathways or cellular mechanisms, have led to enhanced overall survival (OS) and progression-free survival (PFS). Currently, anti-angiogenic drugs including bevacizumab, regorafenib and aflibercept, are approved as a treatment of metastatic stage of CRC, whereas immunotherapy for CRC is still limited to the MSI-H tumors (6).

Classification system of CRC, based on molecular structure, was established to categorize both the tumor and the surrounding tumor microenvironment (TME) through variations in CRC gene expression (7). TME is a dynamic ecosystem that plays a crucial role in the support and progression of tumors. The composition of TME may significantly affect the tumor response to immunotherapy. TME includes different types of cells, e.g., tumor-infiltrating lymphocytes (TILs), tumor-associated macrophages (TAMs), tumor-associated neutrophils (TANs), cancer-associated fibroblasts, natural killer (NK) cells, regulatory T cells and dendritic cells (DCs). There are four main consensus molecular subtypes: CMS1, CMS2, CMS3, and CMS4 (8). Both CMS1 and CMS4 subtypes are characterized by increased immune cells infiltration, while CMS1 tumors, in particular, is identified by enhanced Th1-cell response as well as inflamed and stimulated TME. Whereas CMS4 TME is characterized by being inflamed and highly angiogenic, hence a good target for combination therapy. CMS2 tumors are caused by β-catenin pathway activation, with subsequent dendritic and T-cell exhaustion. Therefore, this subtype of tumors does not elicit anti-tumor immune response. CMS3 tumors are characterized by several metabolic pathways dysregulation such as nitrogen, glucose pentose, fatty acids, etc. (7).

## Tumor microenvironment of colorectal cancer

Tumors are cellular networks characterized as being different and complex with de-differentiated malignant cell types, tumor stem cells, fibroblasts and endothelial and immune cells. TME is a dynamic ecosystem that plays a crucial role in supporting the progression of tumors. Cytotoxic CD8+ T-lymphocytes (CTL) are considered the major defense mechanism against tumor cells, hence T-cell abundance is a decisive and crucial prognostic factor for immunotherapy and chemotherapy response, particularly at the early tumor initiation stage, where an increased activity of T cells has been reported (9). The PD-L1/PD1 axis is identified as an inhibitor of CTL activity in several CRC phenotypes including Mismatch repair deficiency (MMRd)/Microsatellite instability-high (MSI-H) phenotype in which anti-PD1 monoclonal antibodies are highly beneficial in fighting the tumor (10, 11). Another essential type of T-cells highly associated with colorectal tumors is the Regulatory T-cells (Tregs) (12).

Other cell types in the TME include TAMs involved in regulating metastatic phenotype of cancer and modulating growth and invasion of cancer cells (13, 14). Two sub-populations of TAMs have been identified, the pro-tumorigenic (M2) and the anti-tumorigenic (M1) phenotypes, which are characterized by high plasticity (15). TAMs and myeloid-derived suppressor cells (MDSCs) are the most abundant cells in solid tumors including CRC. Moreover, other immune cell types have been identified in the CRC microenvironment, such as NK cells, TANs, eosinophils and mast cells, with variable roles in CRC progression (16, 17). CRC stroma is well-known for its ability to promote tumor-associated blood vessels. Immune cells and fibroblasts supply tumor cells with VEGF (18). Moreover, matrix metalloproteinase and associated proteases, expressed by CAFs, are abundant in TME.

## Autophagy and colorectal cancer

### Autophagy signaling in cancer

Autophagy has a diverse and dynamic impact on cancer cells that can affect both tumor initiation, progression and cancer response to therapy. Recently, vast published data indicate a crosstalk between autophagy-related genes (ATG’s) associated pathways with oncogenes and/or tumor suppressor genes. Indeed, the precise role of autophagy in modulating cancer tumorigenicity is highly complicated and is dependent on the context (19). Several autophagy genes might be involved in switching normal cells to CRC under particular conditions. The first autophagy marker indicated to be involved in colorectal carcinogenesis is LC3 (20). One of the LC3 isoforms, named LC3-II, is overexpressed in CRC cells particularly in advanced stages, compared to normal colon cells (21). Notably, low LC3 level has been interrelated to good CRC prognosis, particularly in advanced stages (22). Moreover, ATG5 and ATG10 showed a major role in CRC progression and chemotherapy resistance in several studies. ATG5 was found to be down-regulated in 95% of CRC cases, and its high expression level indicates lympho-vascular invasion (23). In contrast, ATG10 was upregulated in CRC tissues and increased protein expression of ATG10 was accompanied by tumor lymph node metastasis and invasion (24). Another essential protein implicated in autophagy is the activating molecule in Beclin-1-regulated autophagy (Ambra1) protein encoded by the *AMBRA1* gene. Mutated *AMBRA1* gene was found in a subset of colorectal neoplasms (25). Additionally, Beclin-1 gene, UVRAG gene and *Bif-1* gene were highly correlated with CRC carcinogenesis which is explained in the following sections.

#### Role of autophagy in colorectal cancer initiation

Autophagy is an equilibrating mechanism that promotes anti-malignant mechanism by clearance of unhealthy damaged proteins, DNA abnormalities and reactive oxygen species (ROS). A proper autophagic mechanism is crucial for the mutagen’s elimination and appropriate genomic stability as it avoids the genetic defects accumulation that proceeds to malignant transformation. Thereby, autophagy might act as a tumor-suppressor in the early stages of the tumor. Evidence demonstrates that the tumor-suppressive effect is derived from some ATG-proteins such as Beclin-1, which shows anti-oncogenic properties. Tumor suppressor role of Beclin-1 is validated genetically in breast, ovarian and prostate tumors, as mono-allele deletion of Beclin-1 occurs (26, 27). However, Beclin-1 has a debatable role in CRC in that it promotes tumorigenesis, but may paradoxically inhibit CRC cell growth. Increased Beclin-1 expression was associated with better OS in patients with locally advanced colon carcinomas who received postoperative 5-FU chemotherapy for 6 months (28). Beclin-1 Overexpression in cases with resected stage II and stage III colon carcinomas, who received 5-FU-based therapy was associated with worse OS, denoting a potential effect of autophagy in drug resistance (29).

Moreover, allelic loss of UVRAG, an autophagy component, and attenuation of *Bif-1* expression that both interact with Beclin-1 directly, might be correlated to CRC initiation and development (30). UVRAG protein is needed to form a complex with Beclin-1 to induce autophagy; therefore, the loss of this protein results in impaired autophagy machinery. Similarly, Bif-1 serves to induce autophagy *via* interacting with Beclin-1 and UVRAG.

Autophagy displays an important defense mechanism against pathogens and therefore plays an anticarcinogenic role in combatting viral and bacterial infections. For example, autophagic machinery was shown to effectively eliminate digestive cancer-associated pathogens such as *Streptococcus bovis* (*S. bovis*) that may cause CRC (31). In the same study, using autophagy-deficient *ATG*5-/- cells showed *S. bovis* pathogen survival and enhanced multiplication within the cells (31). The presence of infectious endocarditis of *S. bovis* may be followed by colonic neoplasia in an estimated incidence of 18–62% of cases, even after years of its presentation in the host (32, 33). Similarly, 25 to 80% of *S. bovis* bacteremia cases induce colorectal tumors (34). Despite this, the relationship between CRC and *S. bovis* bacteremia has been underestimated for a long time and is under the controversy of whether this association is a result of gastro-intestinal tumor or the *S. bovis* itself could be the etiology of CRC (35).

#### Role of autophagy in colorectal cancer cell survival and metastasis

In previous studies, autophagy seems to support tumor progression. Autophagy helps tumor cells overcome induced metabolic stress resulting from high proliferative rate, hypoxia and nutrient deprivation due to insufficient blood supply needed by these tumors for proliferation and progression (36, 37). Cancer cells consume more energy and metabolites than normal cells due to their rapid proliferative rate. Both energy and metabolites can be provided to cancer cells by increasing autophagy (38). Autophagy is considered a survival mechanism for cancer cells under hypoxic and metabolic stress conditions to provide them with the energy required for their survival and proliferation (39). In this regard, down-regulation of crucial autophagy proteins level led to restraining cancer growth and reduced oxygen consumption along with the accumulation of abnormal mitochondria, and specifically, autophagy was demonstrated to be essential to promote the growth of *Ras*-driven tumors, including CRC (40). Several *in vitro* studies indicated that gaining autophagy activity in *Ras*-driven cancer cells shows a significant increase in the survival and progression of those cancer cells in several settings of metabolic stress (41).

Besides its critical role in regulating protein turnover and cancer immunogenicity, autophagy has been involved in epithelial-to-mesenchymal transition (EMT), a crucial multistep mechanism needed by tumor cells to metastasize (42, 43). The commonly identified EMT inducer TGFβ is known to induce EMT through the stimulation of SMAD, MAPK, Rho-GTPases and PI3K/AKT (44). During tumor progression, cells that undergo EMT need to stimulate autophagy machinery for their survival and metastases. In this regard, it has been demonstrated that autophagy is essential for EMT activation and cancer cell metastasis in hepatoblastoma cells (45). Similarly, autophagy is needed in TGFβ1-mediated EMT in non-small-cell lung cancer cells (46). In CRC cells and upon using rapamycin, a specific mTOR inhibitor and an autophagy inducer, starvation-mediated autophagy was demonstrated to induce invasion and migration and increase EMT marker expression; and interestingly, this was reverted by *Beclin-1* knockdown (47).

#### Effect of autophagy on cancer stem cells

Cancer stem cells (CSCs) are recognized to promote tumor initiation, progression and contribute to therapy resistance. CSCs drive tumor heterogeneity *via* EMT and inflammatory signaling activation (48). Autophagy is identified to promote the survival and control the pluripotency of CSCs in the TME. IL-17B/IL-17RB signaling induces autophagy, and subsequently, autophagy controls and maintains CSCs homeostasis. Interestingly, TRAF6 is recruited in the cytoplasm by IL-17B, which would induce autophagosome formation through Beclin-1 ubiquitination, thus promoting self-renewal and sphere-forming potential in gastric carcinoma (49). Likewise, IGF-2/insulin receptor signaling controls CSCs stemness and pluripotency through autophagy regulation. In CRC, loss of imprinted gene expression of IGF-2 indicated increased autophagy, leading to higher sphere-forming potential, and increased *CD133* expression, which is a marker of stemness (50).

**FIGURE 1:**
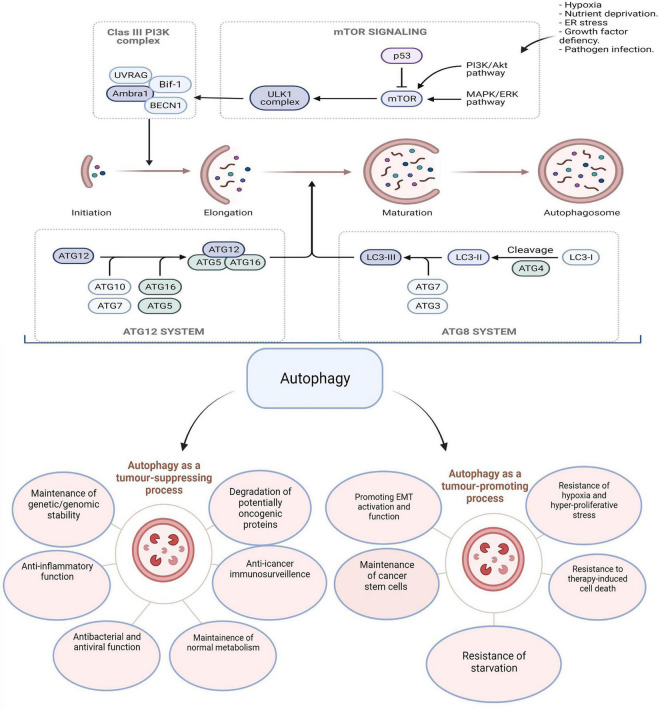
Multiple steps are involved in autophagy machinery: induction, initiation, vesicular expansion, lysosomal fusion, and degradation. Autophagy has contradictory roles in tumorigenesis by either promoting or suppressing depending on the stage of cancer. The figure was modified from Burada et al. (52).

Increased autophagic flux is highly maintained and required by CSCs to promote therapy resistance. In CRC, SOX2 transcriptional factor increases the expression of EMT and *ABCC2* genes and promotes chemotherapy resistance through translocation and activation of β-catenin. Interestingly, SOX2 tends to increase *Beclin-1* expression to induce autophagy and promote chemoresistance. Thus, SOX2-β-catenin/Beclin-1/autophagy pathway is involved in tumor progression and chemotherapy resistance (51). A graphical illustration of the autophagy signaling pathway and its dual role in CRC initiation and progression is displayed in Figure 1.

### Autophagy signaling modulates tumor microenvironment

Autophagy is actively involved in remodeling TME *via* unconventional secretion of several peptides, proteins and hormones that are typically operated and secreted through the conventional secretory system controlled by the endoplasmic reticulum–Golgi pathway (53). Knockdown of autophagy in both stromal cells and cancer cells is associated with a reduction of several cytokines and chemokines release including IL-1β, IL-2, IL-6, IL-8, IL-18, CCL2, CCL20, TNFα, and LIF. Herein, autophagy is capable of modulating tumor growth, metastasis and angiogenesis as well as immune evasion and stemness maintenance, through autophagy-dependent secretion of pro-inflammatory and pro-invasive factors (54–57). Another tumor secretome released in an autophagy-dependent manner includes growth factors (TGF-β1, b-FGF), extracellular matrix proteins (MMP2, MMP9) and the angiogenesis stimulant (VEGFA) (Table 1) (55, 58, 59). Additionally, autophagy deficiency impedes the release and secretion of crucial cytokines and chemokines involved in T cells and DC recruitment, including IFN-*γ*, CXCL9, CXCL10, and CXCL11, thus immune surveillance escape occurred (Table 1) (60).

**TABLE 1:** Summarized list of the crucial autophagy-dependent secretome and inflammatory mediator in TME.

Substances/Secretome	Definition and function
TGF-β1	Transforming growth factor β-1 (TGF-β1) is an important pleiotropic cytokine in wound healing, immunoregulation, angiogenesis and cancer. TGF-β1 isoform is produced by immune cells that exert powerful anti-inflammatory functions.
β-FGF	Beta- Fibroblast Growth Factors (β-FGF) are involved in cell proliferation, differentiation, normal development, wound repair, and angiogenesis. β-FGF is mostly produced by stromal cells in bone marrow, leukemic cells, and T cells. β-FGF is an important regulator in the self-renewal and differentiation of multipotent hematopoietic progenitor cells.
MMP2	Matrix metalloproteinase-2 (gelatinase a); is a type IV collagenase that plays a role in vasculature remodeling, angiogenesis, tissue repair, tumor invasion, inflammation, and atherosclerotic plaque rupture. Also, MMP2 functions as degrading extracellular matrix proteins.
MMP9	Matrix metalloproteinase-9; potentially involved in local proteolysis of the extracellular matrix, leukocyte migration and bone osteoclastic resorption. Also, it cleaves type IV and type V collagen and fibronectin degradation.
VEGFA	Vascular endothelial growth factor-A is involved in angiogenesis, vasculogenesis and endothelial cell growth. As well as it Induces endothelial cell proliferation, promotes cell migration, inhibits apoptosis and induces permeabilization of blood vessels.
IFN-*γ*	Interferon *γ*; Produced mostly by lymphocytes, has antiviral activity, and an important immunoregulatory functions. It acts as an activator of macrophages and has anti-proliferative effects on transformed cells. IFN-*γ* can potentiate the antitumor effects of the type I interferons.
CXCL9	C-X-C motif chemokine 9; is a cytokine that impacts the growth, movement, or involved in the immune and inflammatory response. It acts as a chemotactic for activated T-cells.
CXCL10	C-X-C motif chemokine 10; Chemotactic for monocytes and T-lymphocytes. Binds to CXCR3; Belongs to the intercrine alpha (chemokine CxC) family.
CXCL11	C-X-C motif chemokine 11 is an important chemotactic for interleukin-activated T-cells, neutrophils, or monocytes. CXCL11 induces calcium release in activated T-cells. Also, it is participating in CNS diseases that involve T-cell recruitment.

In contrast, autophagy stimulates the release of specific proteins known as DAMPs (damage-associated molecular patterns) that enhance an immunomodulatory effect by triggering immune cells. Therefore, it enhances the anti-tumor immunity and restricts tumor progression (61, 62).

#### Cross-talk of autophagy and anti-tumor immunity

**FIGURE 2:**
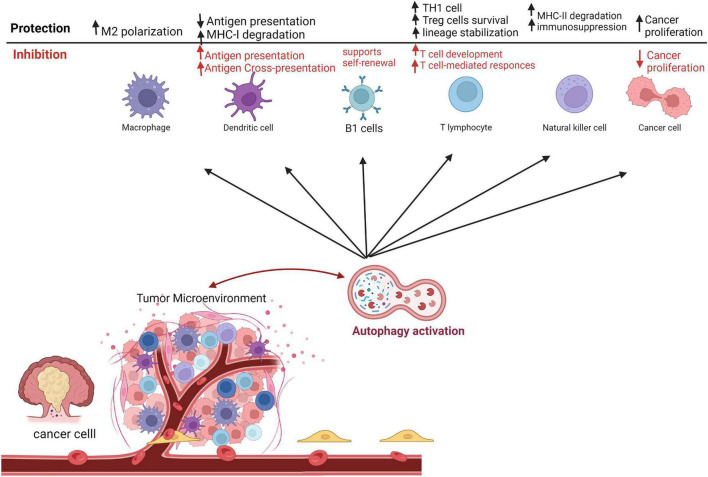
Autophagy roles in immune responses as a tumor-suppressive and tumor protective mechanism in the tumor microenvironment. This figure was modified from Zada et al. (63).

In the age of immunotherapy success to fight cancer, there is an increasing demand to know how autophagy modulation affects the response to anti-cancer medications. Evidence suggested a decline in autophagy levels in aging T lymphocytes, indicating that autophagy inhibition might contribute to hematopoiesis and/or systemic immunity impairment (64). Furthermore, the survival of hematopoietic stem cells and memory T cells are dependent on autophagy (65, 66). In the myeloid compartment, autophagy supports B1 cell self-renewal and provides free fatty acids needed by the differentiating cells (67, 68). Additionally, autophagy has a major influence on the tumor-specific CD8+ T cells (69) and memory T-cells (70). Autophagy has been shown to dictate the degradation of cytolytic granules secreted by cytotoxic CD8+ T cells and NK cells (71, 72). Intriguingly, autophagy has a crucial role in protein degradation, thus allowing antigen-presenting cells (APCs), like DCs, to utilize such proteins as antigens on major histocompatibility complex (MHC)-I and II. The process occurs through three main pathways; namely, exogenous, cross-presentation, and endogenous pathways (Figure 2). Such role was previously reviewed by Koustas et al. (73).

Furthermore, immune suppressor cells have variable responses to autophagy inhibition. For instance, the immunosuppressive effect of Tregs is highly autophagy-dependent (12). Interestingly, it has been indicated that *ATG5 or ATG7* deletion in T cells produces severe tumor implant rejection in the syngeneic mouse tumor model (74). Another published work demonstrated that inhibition of *Beclin-1* gene expression enhances T cells infiltration into the TME (75).

In the developed TME, TAMs, M2 phenotype, are vital in the growth and metastasis of cancer cells, as well as angiogenesis (76). On the other hand, several studies proposed that M1 macrophages inhibit tumor progression (77). Autophagy has been shown to participate in the production and polarization of macrophages. Toll-like receptor-2 (TLR2) deficiency is associated with autophagy inhibition and subsequently results in the biosynthesis of M2-type macrophages, which in turn supports tumor progression (78). In addition, autophagy initiation in TAMs promotes apoptotic cell death, restrains proliferation, and enhances radiosensitivity of CRC (79). Altogether indicated that autophagy in TAM plays an essential role in suppressing cancer (Figure 2).

Furthermore, other native immune cells critically participate in CRC tumorigeneses, such as tumor-associated neutrophils (TANs) and NK cells (Figure 2). For instance, promoting autophagy in TANs enhances the migration and metastasis of cancer cells (80). Analogous outcomes have been reported in other cancer types such as melanoma and renal cell carcinoma (81).

#### Autophagy as a regulator of immune-checkpoints

Additionally, autophagy has an impact on immune tolerance in response to immunotherapy, since immunologic molecules such as indoleamine 2,3-dioxygenase (IDO), Programmed cell death protein 1 (PD-1), and T-lymphocyte-associated protein 4 (CTLA-4) are regulated by autophagy pathways. IDO can inhibit tumor immunity through its inhibitory effects on cytotoxic T-cell responses, DC maturation, and Treg proliferation, thus promoting immune tolerance and tumor development. However, autophagy can inhibit the production of IDO in tumor sites (82, 83). Tumor cell PD-1 interacts with T-cells PD-L1 and serves as an inhibitory checkpoint molecule, preventing tumor cells from being recognized, thus suppressing the antitumor immunity. It has been reported that PD1 inhibits the availability of nutrients to nearby T-cells by interacting with its ligand, inducing autophagy (84). Results from experiments with murine melanoma cells and human ovarian cancer cells suggest that PD-L1-overexpressing cells are more responsive to autophagy inhibitors than cells with weak PD-L1 expression. This finding suggests that autophagy inhibitors may become an important therapeutic tool in PD-L1-overexpressing cancer cells (85). However, further experiments are warranted to explore how PD-L1 signaling and autophagy operate in different cell types, including CRC. This will assist in determining whether anti-PD-L1 therapy combined with autophagy inhibitors will enhance antitumor responses. The CTLA-4 protein is another immune tolerance checkpoint that can be targeted to treat tumors. A cancer-antigen called MAGE-A is associated with CTLA-4 inhibitor resistance and is known to suppress autophagy, suggesting that autophagy induction may be used therapeutically as a way to improve the efficacy of CTLA-4 inhibitors in human melanomas (86). Further experiments are needed to explore cross-talk of autophagy and immune checkpoints in CRC as well. Immune checkpoint therapy for CRC, as a whole, remains unsatisfactory at present. However, there has been renewed interest in examining additional immune checkpoint molecules. New immune checkpoint targets have been identified like the T cell immunoglobulin and mucin domain containing-3 (TIM-3), the V-domain Ig suppressor of T cell activation (VISTA), the T cell immunoglobulin and ITIM domain (TIGIT), and the lymphocyte activation gene-3 (LAG-3) (87–89). Despite an exponential growth in clinical trials for emerging immune modulators, such as anti-LAG-3 antibodies and anti-TIM-3 antibodies, registered on ClinicalTrials.gov, no drugs have yet been approved for clinical use. Despite promising monotherapy results, more effort needs to be integrated toward developing rational combinations of immune-therapy to inhibit cancer growth through non-redundant pathways that work synergistically.

#### Cross-talk of autophagy and endothelial cells

The innermost layer of blood vessels is lined by endothelial cells. In addition to being essential for normal tissue function, new blood vessels also play an important role in cancer pathology. For tumor cells to grow and spread, neovascularization is necessary. Tumor endothelial cells have a multifaceted functional role since they are not only responsible for enhancing angiogenesis, but are also important in immune regulation in the TME (90). Regulatory mechanisms profoundly influence peripheral immune cell recruitment into the TME by acting as significant gatekeepers during cellular transmigration (91–93). Furthermore, tumor endothelial cells act as antigen-presenting cells (APCs), which are associated with T cell activation, proliferation, and priming (92). Furthermore, tumor endothelial cells are required for the development of “tertiary lymphoid structures,” which are associated with the response to checkpoint antibody therapy (94). Other qualities that distinguish tumor endothelial cells from normal endothelial cells are their high proliferation potential and markedly changed gene expression profile (i.e., an increase in pro-angiogenic, extracellular matrix remodeling, and stemness genes), leading to increased secretion of immunomodulatory cytokines and altered cell-surface receptors, e.g., MHC and immune checkpoints (90, 95). It is possible that the tumor endothelial cells phenotype is rooted in an aggressive tumor micro-milieu driven by hypoxia and ROS (96, 97). In clinical practice, chemotherapy combined with angiogenesis inhibitor results in marked enhancement of anti-cancer effects in patients with metastatic CRC (98).

Increasing evidence suggests that autophagy impacts endothelial cell survival, proliferation, migration and angiogenesis. However, whether autophagy regulates angiogenesis positively or negatively is still debated. For instance, according to Du et al., overexpressing *ATG5* induced autophagy in bovine endothelial cells resulting in enhanced formation and migration in those endothelial cells while 3-methyladenine (3-MA) or siRNA targeting *ATG5* reduced angiogenesis (99). A study by Goyal et al. discovered that decorin-induced autophagy provided protection against tumor neovascularization and epithelial death (100). Autocrine VEGF released from endothelial cells and gastrin-releasing peptide (GRP) secreted by tumors promote angiogenesis, endothelial survival, and proliferation of endothelial cells by inhibiting autophagy (101). Moreover, a study carried out by Seon-Jin Lee et al. established that genetically disrupting *Beclin1* can increase tumor growth and angiogenesis in hypoxic environments (102). A broader view suggests that autophagy can influence the angiogenesis process, which is important to tumor growth, by affecting the function and survival of endothelial cells, which has a pro- or anti-tumor effect on CRC.

#### Autophagy and colorectal cancer metabolism

Autophagy is a conserved catabolic process by which various proteins, cytoplasmic constituents and organelles can re-enter the different metabolic processes. Cancer cells altered their metabolism, thus promoting their proliferation, progression, and long-term survival. Cancer cells enhance glucose uptake and metabolize glucose to lactate even when completely functioning mitochondria support the oxidative phosphorylation mechanism, altogether is known as Warburg effect (103). In the normal process, pyruvate kinase (PKM2), the enzyme catalyzing the last step in the glycolytic process, takes control of the glycolytic flux, preventing the excessive accumulation of glycolytic metabolites (104, 105). However, pyruvate kinase (PKM2) enzyme breakdown is enhanced in cancer cells *via* chaperon-mediated autophagy, thus associated with increased accumulation of glycolytic metabolites (106). Also, hexokinase 2 (HK2), rate-limiting enzyme of the glycolytic pathway, is selectively damaged by autophagy in liver carcinoma (107, 108). Therefore, autophagy plays a vital role in cancer metabolism *via* controlling glycolysis at different stages and levels. Warburg effect elevates lactate level in the TME that disturbs the extracellular environmental pH, resulting in autophagy activation (109). For instance, acute acidification of breast cancer cells results in increased expression of *LC3, ATG5, and BNIP3* (110). Therefore, autophagy destructive effect on vital metabolic enzymes may critically influence many features of central metabolism in cancer. Hence, autophagy contributes to malignancy progression and transformation by providing cancer cells with the efficient ability to re-distribute metabolites allowing metabolic rewiring.

Moreover, as a result of starvation, infections, and cancer, glutamine homeostasis is disturbed and the need for exogenous glutamine to promote cell survival and growth is increasing (111). Due to the Warburg effect, glutamine is excessively required to sustain oxidative phosphorylation through its role as a key intermediate in the tricarboxylic acid (TCA) cycle. Furthermore, it is the main nitrogen source for many aminotransferase enzymes involved in the synthesis of nucleotides and non-essential amino acids (112). Glutamine participates in redox homeostasis by contributing to NADH/NADPH synthesis and glutamate synthesis, which is critical for glutathione synthesis (112). Therefore, with such a wide range of glutamine functions, it is critical for some cancers including CRC to ensure an adequate glutamine supply (113). Targeting glutamine transport and metabolism has therefore been a promising approach for treating CRC (113). As soon as glutamine is deficient or lacking, the cells show differential manifestations, including a pronounced decline in ATP and NADH, as well as a significant accumulation of ROS (114, 115). Herein, Autophagy plays an important role in this adaptive response by suppressing glutamine-consuming processes and elevating glutamine content in the body. Macro-pinocytosis is one of the mechanisms by which activated autophagy restores glutamine levels *via* recycling intracellular proteins and extracellular compartments (116). Meanwhile, some reports claim that autophagy plays a crucial role in cancers that escape death with high success rates (117). Upon limitation of exogenous glutamine, inhibition of autophagy in SW620 and SW480 colorectal cell lines resulted in increased apoptotic activity (118). In the same way, chronic activation of mTORC1 may result in severe mTORC1-dependent cell death (later termed glutamoptosis), ultimately inhibiting autophagy (119). In nutrient starvation, autophagy activation is often associated with cell survival. However, over-activating autophagy in specific contexts has shown anti-tumor potential.

#### Role of autophagy in the regulation of hypoxia and oxidative stress in tumor microenvironment

Autophagy plays a pivotal role in helping cancer cells adapt and survive under hypoxic TME. Intriguingly, autophagy promotes the survival of cancer cells through its main effector, hypoxia-inducible factor-1α (HIF-1α), which is mostly the case in solid tumors, specially CRC (120). Tumor cells can endure hypoxia through Beclin1-mediated cytoprotective autophagy by upregulating the transcription of *BNIP3 and BNIP3L* (121). Moreover, BNIP3L/NIX functions as a selective receptor for autophagy that is highly expressed in tumor cells, which is crucial to promote mitophagy under hypoxic TME through NFE2L2/NRF2 transactivation. In addition, cells overexpressing NIX, are more susceptible to acquire glioma stem cell-like properties *via* mTOR/AKT/HIF pathway (122). Under hypoxic conditions, a crucial adaptor protein, FUNDC1, is triggered to eliminate dysfunctional mitochondria. FUNDC1 protein functions critically in autophagy *via* engaging with LC3 protein through LC3 interacting region (LIR) of FUNDC1 (123). Additional form of autophagy regulation under hypoxia occurs *via* HMGB1 signaling through upregulating *YAP* expression in tumor cells. Similarly, ATG5 and ATG12 are stimulated by PAK1 acetylation and PTBP3, respectively, resulting in promoting pro-survival autophagy. Furthermore, an important kinase, PRKCA/PKCα, that regulates hypoxia-mediated autophagy *via* ATG5 and Beclin1, stimulates tumor-initiating cell renewal in CRC (124). Likewise, *YTHDF1* gene is activated by HIF-1α to promote autophagy protective effect through ATG2A and ATG14. Of note, protein phosphatase 2 (PP2A) along with mTOR downstream kinase signaling pathways control the prolyl hydroxylase domain-containing protein 2 (PHD2) phosphorylation to govern and promote HIF-1α mediated autophagy in CRC cells survival (125). Also, *ANKRD37* gene is demonstrated to induce HIF-1α mediated autophagy in hypoxic colon cancer once it translocates to the nucleus (126).

Hypoxia-mediated HIF-1α induction is reported to promote autophagy, thus controlling glycolytic processes to maintain energy supply and cell progression. In this regard and under hypoxic conditions, proline gets metabolized into pyrroline-5-carboxylate (P5C) with the help of proline oxidase (POX) enzyme, which elicits ROS production that promotes protective autophagy mechanism, which is necessary for the survival of HT29 cells (127). Proline oxidase (POX) enzyme role is AMPK-dependent; however, it is controlled in HIF-1α and HIF-2α independent manner (127).

Interestingly, autophagy was demonstrated to restrain oxidative stress-dependent inflammation and promote tumor-suppressor mechanisms. For instance, the transcription activator “BRG1” stimulates autophagosome biogenesis by regulating the transcription of *ATG7, AMBRA1, and Wipi2*, thus attenuating colonic inflammation and CRC development in an oxidative stress-mediated autophagy manner (128).

## Autophagy targeted therapy in colorectal cancer

### Recent and ongoing clinical trials

Despite the controversial and contextual relationship between cancer and autophagy, it is still considered a promising target for treatment, as many shared regulatory pathways of carcinogenesis and autophagy are involved. Some studies demonstrated that autophagy induction is highly correlated to the resistance of cancer cells to chemotherapy, immunotherapy, and radiotherapy *via* directly modulating cancer cell metabolism or diminishing cell death pathway (72, 129–131). Thus, various preclinical and clinical studies have been conducted to develop pharmacological autophagy inhibitors (132). The most recent development of autophagy inhibitors can be known by tracing the clinical trials (Table 2). The most effective targeted therapies recognized in CRC treatment, so far, are anti-angiogenesis such as cabozantinib, apatinib and bevacizumab, and the inhibitors of epidermal growth factor receptor (anti-EGFR) such as cetuximab (133).

**TABLE 2:** Previous and current clinical trials involving hydroxychloroquine (HCQ) in combination with a variety of anti-cancer targeted agents in CRC.

Treatment	Target of the treatment	Phase	Patients number	Status	Outcome	Trial reference number at ClinicalTrials.gov/References
Vorinostat + HCQ	Histone deacetylase (HDAC) inhibitor.	I	72	Active not recruiting	No significant clinical improvement in the safety profile and the progression-free survival.	NCT01023737 (142)
Temsirolimus + HCQ	mTOR inhibitor.	I	40	completed	Safe and tolerable, Significant tumor suppression effect.	NCT00909831 (139)
Temozolomide + HCQ	DNA alkylating agent/induce cell cycle arrest at G2/M.	I	38	completed	Safe and tolerable, beneficial anti-tumor effect.	NCT00714181 (138)
Protein kinase B Akt inhibitor (MK-2206) + HCQ	Akt inhibitor.	I	62	Active not recruiting	Tolerable, minimal anti-tumor activity.	NCT01480154 (140)
HCQ, FOLFOX and bevacizumab.	FOLFOX: chemotherapy that inhibits DNA synthesis. Bevacizumab: VEGF/VEGF receptor inhibitor.	II	38	completed	Increases in autophagy marker LC3 with a complete response rate of 11% but without improved OS in the 28 evaluable patients.	NCT01006369 (98)

For decades, chloroquine has been approved in malaria and arthritis treatment and is currently an inhibitor of autophagy *via* inhibiting the fusion of autophagosomes with lysosomes in the last step of autophagy machinery. Hence, many clinical trials are investigating chloroquine or chloroquine derivatives either alone or in chemotherapy or radiotherapy combinations in patients suffering from different forms of cancers. One trial named CHOICES (Chloroquine and Imatinib Combination to Eliminate Stem cells), a phase II trial, is investigating and comparing the effect of imatinib and hydroxychloroquine combination versus imatinib alone in patients with chronic myeloid leukemia, establishing evidence of autophagy inhibitors concept (134). Apatinib, a tyrosine kinase inhibitor of VEGFR2, has been indicated to stimulate autophagy *via* AKT- mTOR signaling pathway in colon cancer cells (135). Additionally, Cabozantinib is an inhibitor of various kinases responsible for angiogenesis, cell growth and metabolism that showed a major autophagy induction in HCT116 and HT29 CRC cell lines. Notably, cabozantinib in combination with autophagy inhibitors promotes apoptosis in HT29 and HCT116 cells (136). In a study using CRC cell lines, bevacizumab stimulates autophagy as evidenced by punctate patterns of LC3, autophagic vacuoles presence and Beclin-1 accumulation. Autophagy inhibition by targeting *ATG5 and Beclin-1, via* RNA interference or chloroquine, enhances the ability of bevacizumab to induce apoptosis and prevent proliferation, verifying the protective role of autophagy. Similarly, *in vivo* studies using small interfering RNA or chloroquine and bevacizumab combination showed significant inhibition in tumor growth when compared to bevacizumab monotherapy (137).

Of note, a combination of temozolomide and hydroxychloroquine is indicated to be safe and tolerable as well as exerted beneficial anti-tumor effect in phase I trial in patients with solid tumors, including CRC, and in advanced melanoma (138). Similarly, another phase I trial documented the significant efficacy of hydroxychloroquine in combination with mTOR inhibitor temsirolimus in tumor suppression (139). On the other hand, a recent phase I study showed that hydroxychloroquine treatment with AKT inhibitor MK-2206 is tolerable but with minimal anti-tumor activity in solid tumors including CRC (140). As evidenced by multiple instances previously reported, autophagy inhibitors as monotherapy might not be a good treatment choice for cancer therapy (141). Treatment combination of hydroxychloroquine with HDAC inhibitor vorinostat in an ongoing phase I study for patients with advanced renal and colorectal cancers shows no significant clinical improvement in the safety profile and in the patient PFS, indicating a limited benefit of adding hydroxychloroquine (Table 2) (142).

In a study on CRC cell lines, autophagy inhibition by 3-MA showed significant 5-FU-induced apoptosis, thus autophagy might have a crucial role in enhancing response of colon cancer cells treated with 5-FU (143). Likewise, another study using chloroquine, an autophagy inhibitor, in combination with 5-FU showed an enhanced anti-proliferative effect of 5-FU in CRC cells (144). More, inhibiting late-stage autophagy has been demonstrated to enhance the apoptotic cell death activity of the pyrrolo-1,5-benzoxazepines (PBOXs) in human CRC cells (145). Moreover, UAMC-2526 displays inhibitory effects on ATG4. This compound abolishes autophagy in mice bearing colorectal tumors and promotes chemotherapy-induced cell death (146). Recent *in vitro* assays and *in silico* screening has identified a new, important ATG4B inhibitor (S130) that has the ability to interfere with ATG4 proteolytic activity but not with other proteases. Also, S130 is well distributed in tissues *in vivo*, enhances cell death in CRC and reduces the tumor size (147). These findings identify ATG4B as a potential anti-cancer target.

### Challenges and potential solutions of the autophagy targeted treatment

Based on studies and clinical trials described above, it seems that autophagy inhibitors have a different clinical response in cancer therapy. Identification of good biomarkers with suitable pharmaco-dynamic properties that can estimate any change in autophagy, is of the major difficulties facing scientists (148). It remains to be explored whether the limited clinical efficacy of chloroquine is correlated with its lack of specificity in inhibiting autophagy. In fact, both chloroquine and hydroxychloroquine are non-selective autophagy inhibitors which are evident by their role in the reduction of nutrient scavenging (149). This diminished targeted delivery results in plummeting the bioavailability of the drugs. However, hydroxychloroquine is characterized by higher bioavailability compared to chloroquine. Moreover, both drugs have been identified to modify the pH of tumors, hence resulting in bioavailability modulation of different cytotoxic drugs when used in combination (150). Furthermore, frequent use of chloroquine has been identified for a long time to elicit renal failure (151). Noteworthy, both hydroxychloroquine and chloroquine could affect pacemaker channels and voltage-gated Na^+^, Ca^2 +^, and K^+^ ion channels in the heart, leading to serious dysrhythmias.

In this regard, there is an urgent need for novel safe autophagy inhibitors with selective targets and a good bioavailability; properties that many proposed drugs failed to reach. One of the major advancements in the field is the discovery of Lys05, a dimeric form of chloroquine, which shows higher accumulation capabilities in the lysosome. Also, Lys05 has been identified to exert potent monotherapy anti-tumor activity in both *in vitro* and preclinical mouse models with limited toxicity in the treated mice. Of note, Lys05 potent characteristic in autophagy inhibition is dependent on C7-Chlorine, bivalent aminoquinoline rings and a short tri-amine linker (152).

Recently, new druggable autophagy target proteins have been established, including Vps34 (or class III PI3K) and Beclin-1. Notably, both proteins are involved in the early autophagy initiation process. A kinase inhibitor, SAR405, inhibits both Vps34 and Vps18, thus diminishing the lysosomal function *via* disturbing the vesicle trafficking between the lysosome and the late endosome. Further, SAR405 has been found to prevent mTOR- and starvation-dependent stimulation of autophagy (153).

Another druggable protein for autophagy modulation which has been recently proposed is the serine/threonine kinase ULK1/ATG1. Identification of small-molecule SBI-0206965, a potent ULK1 inhibitor, was happened through cell-based screen. This inhibitor was found to be high *in vitro* selective for ULK1 kinase as well as suppressed phosphorylation events mediated by ULK1 kinases. Markedly, SBI-0206965 anti-tumor effect has been evidenced *in vivo* as it showed potent tumor inhibition when combined with mTOR inhibitors, hence allowing it for use in the clinic (154). However, a major limitation of this molecule is that it could affect the activity of other kinases including JAK3, FLT3, FAK, and Src.

## Conclusion and perspectives

A large number of proteins involved in the complex process of autophagy, which appears to play a significant role in all stages of carcinogenesis as it impacts tumor progression, initiation and metastatic capacity. Although the role of autophagy is not fully understood in cancer, it is thought to play both a promoting and inhibiting role depending on the context. Thus, it is imperative to identify how these apparently paradoxical roles of autophagy are regulated in CRC, and to constitute an overall view of the mechanisms that enable autophagy to play one role, not the other.

Autophagy modulates the effect of hypoxia and oxidative stress, regulates metabolism, promotes cancer stem cells and constrains the surveillance of immune cells to support cancer progression. The development of several therapeutic agents that modulate autophagy in CRC has led to promising results, supporting their use to enhance the action of other medications. Currently, autophagy inhibitors used in cancer therapy are limited to hydroxychloroquine and chloroquine that require close monitoring, when used for a prolonged period, for hepatic and renal adverse effects. Therefore, there is an urgent need for more translational and basic research to clarify the intricate role of autophagy, and to resolve unanswered questions about the enhanced efficacy of autophagy-targeted cancer therapy. Notably, there is an increased interest in personalized cancer treatment by joining the TME modulation status with advanced technology to explore the alteration in cancer progression. This will hopefully propose a major success in cancer therapy.

## Author contributions

EM and MS-A: conceptualization. EM: writing—original draft preparation and visualization. JT, NS, and MS-A: writing—review and editing. All authors have read and agreed to the published version of the manuscript.
